# Quantifying the Emergence of Dengue in Hanoi, Vietnam: 1998–2009

**DOI:** 10.1371/journal.pntd.0001322

**Published:** 2011-09-27

**Authors:** Hoang Quoc Cuong, Nguyen Tran Hien, Tran Nhu Duong, Tran Vu Phong, Nguyen Nhat Cam, Jeremy Farrar, Vu Sinh Nam, Khoa T. D. Thai, Peter Horby

**Affiliations:** 1 Oxford University Clinical Research Unit, Wellcome Trust Major Overseas Unit, Hanoi, Vietnam; 2 Pasteur Institute, Ho Chi Minh City, Vietnam; 3 National Institute of Hygiene and Epidemiology, Hanoi, Vietnam; 4 Preventive Medicine Center, Hanoi, Vietnam; 5 Centre for Tropical Medicine, Nuffield Department of Clinical Medicine, University of Oxford, Oxford, United Kingdom; 6 Division of Infectious Diseases, Tropical Medicine and AIDS, Academic Medical Center, Amsterdam, The Netherlands; 7 Center for Infection and Immunity, Academic Medical Center, University of Amsterdam, Amsterdam, The Netherlands; Pediatric Dengue Vaccine Initiative, United States of America

## Abstract

**Background:**

An estimated 2.4 billion people live in areas at risk of dengue transmission, therefore the factors determining the establishment of endemic dengue in areas where transmission suitability is marginal is of considerable importance. Hanoi, Vietnam is such an area, and following a large dengue outbreak in 2009, we set out to determine if dengue is emerging in Hanoi.

**Methods and Principal Findings:**

We undertook a temporal and spatial analysis of 25,983 dengue cases notified in Hanoi between 1998 and 2009. Age standardized incidence rates, standardized age of infection, and Standardized Morbidity Ratios (SMR) were calculated. A quasi-Poisson regression model was used to determine if dengue incidence was increasing over time. Wavelet analysis was used to explore the periodicity of dengue transmission and the association with climate variables. After excluding the two major outbreak years of 1998 and 2009 and correcting for changes in population age structure, we identified a significant annual increase in the incidence of dengue cases over the period 1999–2008 (incidence rate ratio  = 1.38, 95% confidence interval  = 1.20–1.58, p value  = 0.002). The age of notified dengue cases in Hanoi is high, with a median age of 23 years (mean 26.3 years). After adjusting for changes in population age structure, there was no statistically significant change in the median or mean age of dengue cases over the period studied. Districts in the central, highly urban, area of Hanoi have the highest incidence of dengue (SMR>3).

**Conclusions:**

Hanoi is a low dengue transmission setting where dengue incidence has been increasing year on year since 1999. This trend needs to be confirmed with serological surveys, followed by studies to determine the underlying drivers of this emergence. Such studies can provide insights into the biological, demographic, and environmental changes associated with vulnerability to the establishment of endemic dengue.

## Author Summary

Dengue is the most common vector-borne viral disease of humans, causing an estimated 50 million cases per year. The number of countries affected by dengue has increased dramatically in the last 50 years and dengue is now a major public health problem in large parts of the tropical and subtropical world. It is of considerable importance to understand the factors that determine how dengue becomes newly established in areas where the risk of dengue was previously small. Hanoi in North Vietnam is a large city where dengue appears to be emerging. We analyzed 12 years of dengue surveillance data in order to characterize the temporal and spatial epidemiology of dengue in Hanoi and to establish if dengue incidence has been increasing. After excluding the two major outbreak years of 1998 and 2009 and correcting for changes in population age structure over time, we found there was a significant annual increase in the incidence of notified dengue cases over the period 1999–2008. Dengue cases were concentrated in young adults in the highly urban central areas of Hanoi. This study indicates that dengue transmission is increasing in Hanoi and provides a platform for further studies of the underlying drivers of this emergence.

## Introduction

Dengue is caused by infection with one of four genetically related but serologically distinct dengue virus serotypes, which are transmitted by the bite of an infected female *Aedes* mosquito. It is the most common vector borne viral disease of humans with an estimated 50 million infections every year and around 3.6 billion people living in areas at risk [1], [2]. Over the past 50 years dengue has spread inexorably, with 9 countries reporting dengue transmission prior to 1970 compared to over 124 now, and incidence having increased 30 fold [3]. There are reasons to believe that the expansion of dengue will continue. Whilst the geographic range of *A. aegypti*, the principle urban vector of dengue, has shrunk in some areas, notably the Mediterranean, North America and Australia, it has expanded in Asia and has re-invaded large parts of South America following eradication attempts in the 1950's and 60's [4]. Meanwhile, the geographic range of *Aedes. albopictus*, a secondary vector of dengue, has expanded dramatically [5]. Both *Aedes* vectors are adapted to peridomestic urban habitats that are expected to burgeon over the next four decades, with the urban populations of Africa and Asia predicted to treble and double respectively [6]. *A. albopictus* is also well adapted to rural and temperate environments, and although dengue has been perceived as a predominantly urban disease, the scale and potential for rural dengue transmission is increasingly being recognized [7], [8]. Southeast Asia is at the epicenter of this global dengue outbreak, accounting for 70% of global dengue morbidity and mortality, and is a region with substantial potential for further expansion [8], [9]. Preventive interventions are currently limited largely to vector control but substantial efforts are being made to develop a vaccine.

Dengue epidemiology is a determined by a complex interaction of vector, pathogen, and host biology; macro and microclimate; the physical environment; and social factors [10], [11]. Dengue has been extensively studied in hyper-endemic areas but less work has been done in areas at the margins of transmission, where opportunities may exist to characterize the process of dengue emergence and assess the factors associated with transmission and disease severity in a less complex environment. The development of dengue vaccines is an added imperative to understand dengue epidemiology at various intensities of transmission in order to predict the impact of different vaccination strategies in different epidemiological contexts. Hanoi is the capital of Vietnam and has a sub-tropical climate, with distinct seasons and cool winters. Hanoi experiences annual seasonal dengue outbreaks with little or no transmission in the intervening months. Increasing numbers of notified dengue cases have led to concerns that dengue is ‘emerging’ in Hanoi, and in 2009 Hanoi experienced its largest ever-recorded outbreak of dengue. In this study, we set out to investigate if dengue is emerging in Hanoi by quantifying changes in disease incidence and the age of notified cases over the 12-year period 1998 to 2009 after adjusting for demographic changes. To explore disease dynamics we describe the temporal patterns of dengue incidence and its association with local climate variables. This work was conceived as the first step in studying the process of dengue emergence in Hanoi and as a framework for developing further studies of the determinants of dengue infection and disease risk in a low transmission setting.

## Materials and Methods

### Ethics statement

The study used public health surveillance data routinely collected through the Ministry of Health notifiable diseases surveillance program. The study did not use patient medical records and all data were analyzed anonymously. The analysis of the surveillance data was approved by the National Institute of Hygiene and Epidemiology, a specialized institute of the Vietnam Ministry of Health.

### Setting

Hanoi is Vietnam's capital and its second largest city. It is located in the north of the country in the low lying and densely populated Red River delta. In the 2009 decennial census, the population of Hanoi was estimated at nearly 6.5 million, with a population density of 1943 persons per km^2^. In contrast to South Vietnam, which is always hot but with dry and rainy seasons, North Vietnam has four distinct seasons with hot and humid summers, receiving the majority of rainfall, and cool and relatively dry winters.

### Study design and data sources

Dengue is one of 24 infectious diseases in Vietnam for which there is monthly mandatory reporting from all administrative levels, which, in increasing size, are communes, districts, provinces, and regions. The detection and reporting of dengue cases follows the Ministry of Health 1999 guidelines on surveillance, diagnosis and treatment of dengue [12]. The case definition for suspected clinical dengue infection is: 1.) fever or history of acute fever, lasting from 2 to 7 days; and 2.) headache, myalgia, arthralgia, and rash. Prior to 1999 suspected dengue cases were reported to the MoH according to the WHO surveillance case definition. If a patient fulfills the clinical dengue case definition, a single blood sample may be taken and sent to the National Institute for Hygiene and Epidemiology for the detection of anti-dengue virus specific immunoglobulin (Ig) M antibodies by an in-house, M-antibody capture enzyme linked immunoassay (MAC-ELISA) as previously described [13]. ELISA results were only collected through the surveillance system from 2002 onwards.

Anonymous individual case reports of all clinically diagnosed dengue cases of any disease severity in Hanoi residents reported through the public health surveillance system were obtained from Hanoi Preventive Medicine Center (PMC) from1st January 1998 to 31^st^ December 2009. Data available on every case were gender, commune and district of residence, and month and year of diagnosis. Age was missing for two subjects only.

Age stratified population estimates for Hanoi by district were derived as follows. Data for 1999 and 2009 were obtained directly from the decennial national Population and Housing Census' conducted by the General Statistic Office of Vietnam (GSO. http://www.gso.gov.vn). Age stratified population estimates for 2000–2008 were obtained from GSO and back-adjusted based on the results of the 2009 census. Since age stratified population estimates for Hanoi for 1998 were not available, we derived these by applying the age structure from the 1999 census to the estimated 1998 population. In 2008 a large province adjacent to Hanoi became a new district of Hanoi. This administrative change increased the population of Hanoi by more than 3 million from 3.2 to 6.5 million. All analyses presented here are restricted to the 14 Hanoi districts that remained unchanged throughout the 12-year study period.

Daily records of total rainfall; mean wind velocity; mean, maximum and minimum temperature; and relative humidity from a central Hanoi meteorology station were obtained from the National Centre for Hydrometeorological Forecasting (http://www.nchmf.gov.vn/web/en-US). Analysis of meteorological data used total monthly rainfall, the monthly mean of daily mean temperatures, the monthly mean of daily mean wind velocity, and the monthly mean of daily mean relative humidity.

### Statistical methods

To adjust for the potential confounding effect of changes in the age structure of the population of Hanoi over the study period, we applied age specific incidence rates for each year to a standard population (direct age standardization) [14]. The standard population was the population estimated by the 2009 Population and Housing Census. This provided age-adjusted annual estimates of the incidence and the median and mean age of notified dengue cases.

To determine if dengue incidence was increasing over time, a quasi-Poisson regression model was used with the annual count of dengue cases as the outcome variable, year as the independent variable, and (log-transformed) mid-year population size as an offset. The age adjusted count per year, rather than per month, was used since monthly counts are non-independent because of seasonal patterns in dengue incidence, and monthly counts would therefore not fulfill the Poisson assumption of independence of outcomes. A quasi-Poisson model was used as the data were over-dispersed. Linear regression was used to test whether there was a linear time trend in the median age of infection. Statistical analysis was conducted in R 2.9.0 (R Foundation for Statistical Computing, Vienna, Austria).

Standardized Morbidity Ratios (SMR) for the 2009 dengue outbreak were calculated using the age-stratified population distribution from the 2009 census and age-stratified dengue incidence in Ba Dinh District in 2009 as the reference population (SMR = 1). SMR's were displayed using the statistical software R 2.9.0 (R Foundation for Statistical Computing, Vienna, Austria).

### Wavelet analyses

Wavelet analysis was used before to explore the periodicity of dengue transmission and the association with climate variables. Wavelet analysis is especially suited to time series where the average, variance, or relationship with co-variates change over time (i.e. non-stationary), and provides the possibility to explore linkages between multiple non-stationary time series [15], [16]. Dengue periodicity was investigated using a continuous wavelet transformation, which decomposes the time series into time-frequency components. Wavelet coherency was performed to quantify associations between monthly dengue cases and local meteorological covariates. Wavelet phase analysis and cross-correlation function (CCF) were used to quantify the lag period over time and mean lag period of a specific frequency (i.e. annual cycle), respectively. Due to the large outbreaks at both ends of the time series (i.e.1998 and 2009) accompanied with low numbers of notified cases in the intervening years, the aggregated dengue time series were log transformed and normalized in order to homogenize the variance prior to analyses. The Morlet wavelet was used and performed in Matlab software. All significance levels were based on 1000 bootstrapped replications.

## Results

### Annual incidence and median age of infection of notified dengue cases

**Figure 1:**
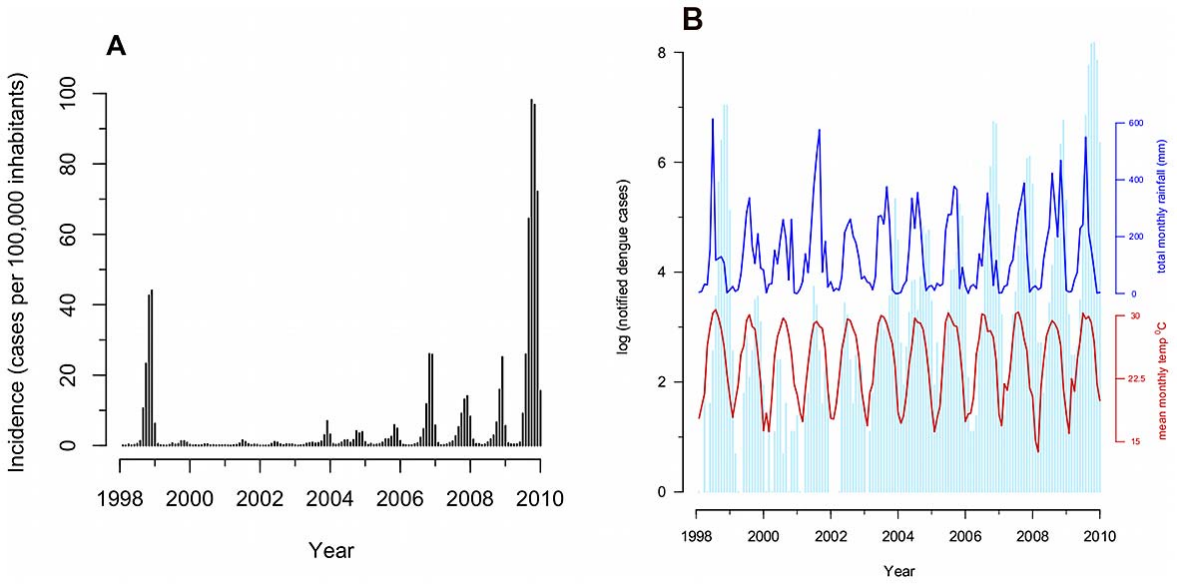
Notified dengue cases Hanoi, 1998–2009. (A) Incidence of notified dengue cases per 100,000 persons, 1998-2009. (B) Log number of notified dengue cases, 1998–2009. Red line is the mean monthly temperature in degrees centigrade. Blue line is the total monthly rainfall in millimeters.

Between 1998 and 2009, 25983 dengue cases were notified to Hanoi PMC. Large outbreaks were observed in 1998 and 2009, with smaller annual outbreaks in intervening years (figure 1a). The annual outbreaks typically begin around July, peak in October, and then decrease towards the end of the year, corresponding with the wet and hot periods (figure 1b).

In the quasi-Poisson model there was no statistically significant yearly increase in age adjusted incidence when all years were included (table 1). Since dengue characteristically produces large epidemics interspersed by many years, these unusually large epidemic years may mask underlying trends in the data. Therefore we also ran the quasi-Poisson model with the two epidemic years of 1998 and 2009 excluded. In this analysis of years 1999-2008, the age adjusted dengue incidence increased 1.38 fold annually (table 1).

**Table 1:** Estimated yearly change in dengue incidence based on quasi-Poisson regression (age adjusted).

Time period	Parameter Estimate (log rate ratio)	Standard error (of log rate ratio)	P-value	Estimated incidence rate ratio	95% CI of incidence rate ratio
1998–2009	0.23	0.16	0.18	1.25	0.92–1.71
1999–2008	0.32	0.07	0.002	1.38	1.20–1.58

**Figure 2:**
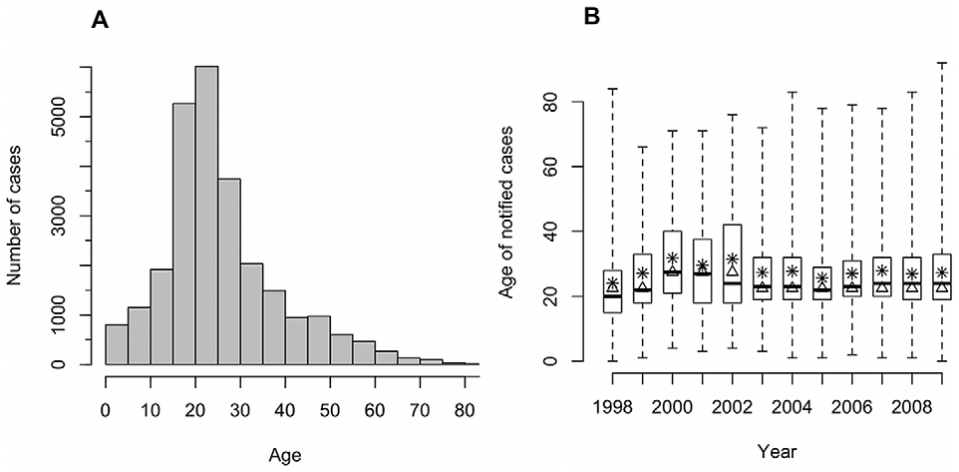
Age of notified dengue cases, 1998–2009. (A) Histogram of age of notified cases, all years combined. (B) Box and whisker plot of unadjusted age of notified dengue cases by year. The black bar denotes the median, the open box the upper and lower quartiles, and the whiskers the maximum and minimum values. The open triangles denote the median age of notified cases after age standardization to the 2009 census data. The T symbol denotes the mean age of notified cases after age standardization to the 2009 census data.

The unadjusted median and mean age of notified cases were 23 and 26.3 years respectively, and 53% of cases were male. The majority of notified cases (85%) occurred in individuals aged over 15 years (figure 2A). Although the average age of notified dengue cases increased over the study period, from a median (mean) age of 20 (22.4) years in 1998 to 24 (26.8) years in 2009, this was not statistically significant after adjusting for changes in the population age structure (figure 2B).

### ELISA results

IgM ELISA results were available from 3678 cases notified between 2002 and 2009. The proportion of cases with an available ELISA result varied from 68.2% in 2002 to 4.5% in 2009. The overall, proportion of ELISA tests that were anti-dengue virus specific IgM positive was 23.7%, ranging from 13.6% in 2006 to 49.8% in 2003. The lowest annual test positivity rates were observed in 2006 (13.6%), 2007 (17.6%), and 2008 (16.1%).

### Standardized Morbidity Ratios

**Figure 3:**
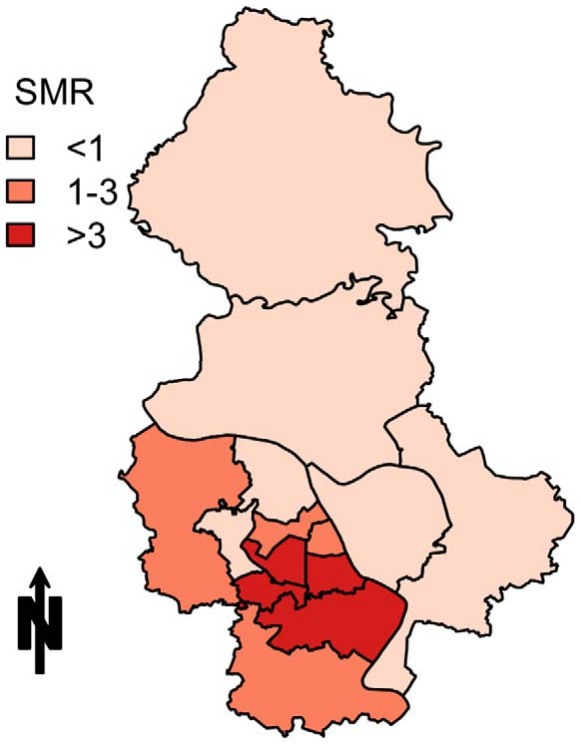
Standardized Morbidity Ratios for notified dengue cases in 14 central districts of Hanoi, 2009.

Dengue SMRs were highest in the central districts of Hanoi, with the districts with an SMR greater than three all being highly urbanized and densely populated areas in the centre of the city (figure 3).

### Periodicity of dengue cases and the association with local climate variables

**Figure 4:**
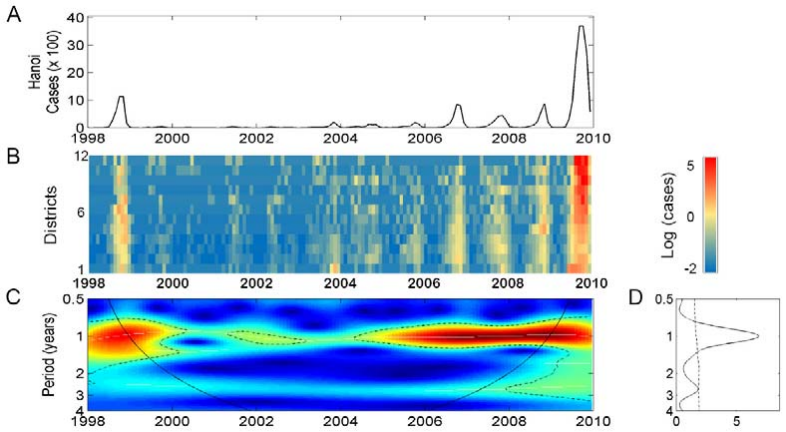
Wavelet analyses of dengue time series with monthly data. (A) Cases per month (x100) in Hanoi from 1998 to 2009. (B) Log transformation of cases in 14 central districts of Hanoi. (C) Continuous wavelet transformation of the aggregated time series. Colors code indicate increasing intensity, from blue to red; black broken lines show statistically significant areas (threshold of 5% confidence interval); the curved black solid line delimits the area within which inferences are robust because of sufficient time series data (the cone of influence), (D) Power wavelet spectrum solid line: the mean spectrum; dotted line: threshold value of 5%.

Continuous wavelet analysis of the aggregated time series showed a significant annual cycle of dengue transmission (figure 4C). Furthermore, a continuous multi-annual band (2–3 years period) was detected, however it did not reach statistical significance in the wavelet power spectrum (figure 4D). Similar dengue periodicity was observed among all districts (figure 4B).

**Figure 5:**
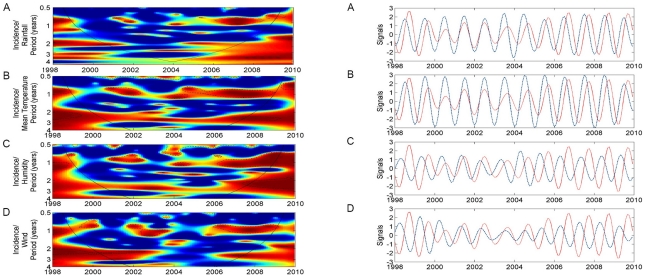
Wavelet coherency and phase analyses between dengue notifications and local climatic variable time series. (A) Rainfall, (B) Mean temperature, (C) Humidity and (D) Wind velocity. The left panel represents the wavelet coherency in which blue and red indicate low and high coherence respectively. The black broken lines show α = 5% significance level. The curved black solid line delimits the area within which inferences are robust because of sufficient time series data (the cone of influence), The right panel represents the oscillations of the time series in the phase analysis. Red lines: dengue time series, blue lines: climatic variables.

All local climatic variables show a statistically significant association with dengue in the 1-year mode (figure 5, left panel). Although significant associations were also observed in the multi-annual band (2–3 years), it should be interpreted with caution since the 2–3 year cycle of dengue cases is itself not significant (figure 4C). The time series oscillations show variability over time in both the lag period and the strength of association (figure 5, right panel). On average, a delay of 1–2 months was observed for rainfall and mean temperature whereas a 4–5 months delay was seen for humidity and wind velocity (figure 5, right panel).

## Discussion

In Hanoi dengue transmission demonstrates clear annual cycles that are associated with a lag of around two months with seasonal increases in mean temperatures and rainfall. This pattern reflects climate driven changes in vector abundance, survival, and biting frequency; and the time it takes for a newly infected mosquito to become infectious to humans (the extrinsic incubation period) [17]–[20]. We observed a pattern of high wind speeds being associated with periods of low dengue notifications, an association noted by other authors [21], [22]. Although it is not established whether this association is causal, high wind speed could conceivably interfere with normal mosquito movements and biting behaviors.

Large outbreaks occurred in 1998 and 2009, and wavelet analysis indicated possible 2–3 years periodicity. A 2–4 years cycle has been described in Thailand, Cambodia, and South Vietnam. Moreover, a meta-cycle between 8–10 years has been observed in Thailand [23]–[28]. This cycle of larger multi-year outbreaks superimposed on an annual cycle is a characteristic feature of dengue epidemiology that has been attributed to various models of serotype-host interactions, and a possible influence of multi-year climate oscillations [10], [23]–[25], [29]–[33]. With a peak of 384 total notified cases per 100,000 persons in 2009, Hanoi has a much lower dengue incidence than other parts of Southeast Asia. Ho Chi Minh City in South Vietnam had around 199 hospitalized cases per 100,000 in 2009, whilst in Thailand and Cambodia the rates of symptomatic dengue infection in children <15 years are estimated at around 24/1000 and 41/1000 respectively [34], [35]. Yet despite these large differences in incidence, similar temporal patterns are observed in Hanoi, suggesting that the intensity of dengue transmission in Hanoi may be sufficient to replicate the inter-subtype dynamics observed in higher transmission areas [29]. This will have relevance for understanding how dengue vaccination may influence intrinsic pathogen-host dynamics in settings with different intensities of transmission [10], [32], [36].

Excluding the two major outbreaks (in 1998 and 2009), the annual age adjusted incidence of notified dengue increased significantly. However, over the same period the average age of infection has not decreased, as would be expected if the per capita rate of infection in susceptible people (the force of infection) was increasing [37]. This apparent discordance could arise through several mechanisms: 1) the recorded increase in incidence may be an artifact of improved case detection and reporting; 2) unmeasured demographic changes, such as an influx of unregistered adult workers, may increase the average age of the population beyond that captured by census and population estimates; 3) susceptible adult in-migration from areas of low dengue risk may increase the relative abundance of susceptible adult individuals; 4) the force of infection has declined resulting in fewer infections and an increase in the median age of infection, but this has resulted in an increase in the incidence of clinical cases (as opposed to infections) since adults are more likely than children to suffer clinically apparent illness as a result of primary dengue infection [38], [39]. It is not possible to distinguish between these scenarios on the basis of passively notified clinical dengue illness alone, and population-based studies with carefully monitored demographics and serial seroepidemiology are needed.

The average age of notified dengue cases in Hanoi is high and 85% are aged over 15 years. Although the average age of clinical cases in Thailand has increased over recent years, the clinical burden of dengue in much of Southeast Asia still primarily falls on children aged less than 15 years [27], [35], [37]. Dengue does however predominantly affect adults in several Southeast Asia countries including Malaysia, Sri Lanka, and Singapore, and dengue is an adult problem in much of the Americas [38], [40]–[45]. The most important influences on geographical differences in the age group predominantly affected are likely to be the local intensity of dengue transmission and the time since dengue was (re)introduced into the population [46]. The high average age of notified dengue cases in Hanoi may reflect a relatively low force of infection but may also reflect relatively recent introduction of dengue into Hanoi such that the adult population has not yet acquired multitypic immunity [46].

Our study is limited by its reliance on passively notified cases of clinically apparent dengue infection. Only a small proportion of all notified cases were dengue IgM positive and the lowest IgM test positivity rates were in 2006, 2007, and 2008. More cases were notified in each of these three years than in any other years except the outbreak years of 1998 and 2009. If the size of the 2006–2008 outbreaks were inflated by the reporting of an unusually large number of non-dengue illnesses, the increase in dengue incidence we have identified may be false. The data reported here are of illness episodes that met the surveillance case definition for dengue, which includes both fever and rash. Measles also presents with fever and rash, and although included in the Expanded Program of Immunization (EPI), there was a large national epidemic in 2009 [47]. However, the peak of this measles epidemic was in February 2009 and was clearly distinct in timing from the dengue epidemic that peaked in October 2009 [47]. Rubella is not included in the EPI and may also be confused for dengue. The occurrence of a large rubella outbreak in Hanoi in 2011 suggests that a large rubella outbreak was unlikely to have occurred in the 5 years prior to 2011 [48]. Chikungunya has not yet been detected in Vietnam. Since dengue often presents as a non-specific febrile illness that is difficult to distinguish clinically from a large range of other illnesses, it is also probable that the reported cases we have used in this analysis significantly under-estimate the true number of dengue infections.

Since anti-dengue virus IgM is not detectable until 3–5 days after infection, and is frequently negative in secondary dengue infections, asays to detect anti-dengue virus IgM antibodies have limited sensitivity (perhaps <20%) when used alone to diagnose dengue infections during the acute illness [49], [50]. Consequently a single negative anti-dengue virus IgM ELISA from an acutely ill patient with clinically suspected dengue is not very helpful in excluding dengue infection. There are also limitations to the specificity of a positive dengue IgM ELISA. IgM ELISA's can remain positive for three months or longer after initial dengue infection, therefore febrile illnesses following dengue infection may be incorrectly diagnosed as dengue on the basis of persisting anti-dengue IgM [50]. False positive results are also possible as a result of cross-reactivity with anti-bodies to Japanese Encephalitis (JE) following infection or vaccination. However JE in Vietnam is predominantly a low-incidence rural problem and the immunization programme has targeted children in higher risk rural Provinces [51]. Therefore it is unlikely that a significant number of dengue IgM positive cases in Hanoi are false positives due to cross-reaction with JE antibodies. Given these limitations, interpretation of single dengue IgM results is difficult, and other diagnostic approaches, such as combined NS1 antigen and IgM assays, should be considered to augment and aid interpretation of clinical surveillance data [52].

As dengue disease severity is modulated by age, prior dengue exposure history, and the infecting dengue serotype, the incidence of clinically apparent dengue cases is unlikely to have a linear relationship with transmission intensity [38], [39], [53]. Therefore data on the age specific incidence of clinical illness cannot be used to directly infer the age specific risk of infection. Seroepidemiological studies are required for this and should be conducted as part of national surveillance programs. Public health authorities in Vietnam have the impression that dengue is an emerging health problem in Hanoi. Although our analysis supports that conclusion, there is a need for population-based seroepidemiology studies that remove any residual confounding by unmeasured demographic factors, and give an unbiased estimate of the age and serotype specific force of infection. The interpretation of such studies will be improved by the recent development of assays that are able to distinguish the original infecting serotype in secondary dengue infections [36]. If the current extensive efforts to develop a dengue vaccine are successful, there exists the exciting potential that appropriately targeted and timed vaccination in areas of low and intermittent transmission may be a cost effective intervention to prevent annual outbreaks or even prevent the (re)establishment of dengue. Such interventions will require a thorough understanding of the dynamics of dengue in such areas.
